# Design and Development of a Real-Time Pressure-Driven Monitoring System for *In Vitro* Microvasculature Formation

**DOI:** 10.3390/biomimetics10080501

**Published:** 2025-08-01

**Authors:** Gayathri Suresh, Bradley E. Pearson, Ryan Schreiner, Yang Lin, Shahin Rafii, Sina Y. Rabbany

**Affiliations:** 1 DeMatteis School of Engineering and Applied Science, Hofstra University, Hempstead, NY 11549, USA; 2 Division of Regenerative Medicine, Hartman Institute for Therapeutic Organ Regeneration, Ansary Stem Cell Institute, Department of Medicine, Weill Cornell Medicine, New York, NY 10065, USArps2001@med.cornell.edu (R.S.);; 3 Department of Surgery, Weill Cornell Medicine, New York, NY 10065, USA

**Keywords:** microfluidics, pressure-monitoring, software, Arduino, real-time monitoring, vascularization, tubulogenesis, flow, pressure

## Abstract

Microfluidic platforms offer a powerful approach for ultimately replicating vascularization *in vitro*, enabling precise microscale control and manipulation of physical parameters. Despite these advances, the real-time ability to monitor and quantify mechanical forces—particularly pressure—within microfluidic environments remains constrained by limitations in cost and compatibility across diverse device architectures. Our work presents an advanced experimental module for quantifying pressure within a vascularizing microfluidic platform. Equipped with an integrated Arduino microcontroller and image monitoring, the system facilitates real-time remote monitoring to access temporal pressure and flow dynamics within the device. This setup provides actionable insights into the hemodynamic parameters driving vascularization *in vitro*. In-line pressure sensors, interfaced through I2C communication, are employed to precisely record inlet and outlet pressures during critical stages of microvasculature tubulogenesis. Flow measurements are obtained by analyzing changes in reservoir volume over time (dV/dt), correlated with the change in pressure over time (dP/dt). This quantitative assessment of various pressure conditions in a microfluidic platform offers insights into their impact on microvasculature perfusion kinetics. Data acquisition can help inform and finetune functional vessel network formation and potentially enhance the durability, stability, and reproducibility of engineered *in vitro* platforms for organoid vascularization in regenerative medicine.

## 1. Introduction

Microfluidics has emerged as a groundbreaking technology offering the capability of recapitulating macroscale physiological conditions on a small scale *in vitro*. By offering precise manipulation of fluids, microfluidics creates organ-on-chip platforms that facilitate the study of cellular behavior and disease pathogenesis [1,2,3,4]. Newly developed platforms are cost-effective and enable fine microscale control over physical and chemical parameters, allowing for a wide range of applications [1,2,4,5,6].

In pursuit of generating functional *in vitro* vascular networks, a promising approach involves reactivating endothelial cells (ECs) using the embryonic-restricted ETS variant transcription factor 2 (ETV2). Expressed during gestational vasculogenesis, ETV2 effectively “Resets” Vascular Endothelial Cells (R-VECs), enabling them to regain the adaptability necessary to form flexible, tube-like endothelial layers reminiscent of native vasculature when embedded within a 3D fibrin scaffold [7,8,9]. The resultant self-organized, stable, and multilayered networks are capable of perfusing blood, adapting to the microenvironmental signals emanating from the organoid or tumoroid that they surround [9]. The development of vascularized organoids will not only allow the deciphering of pathways that choreograph tissue repair but also enable manufacturing of mini organs for regenerative medicine and facilitate their long-term in vivo engraftment [1,5,6]. These innovative models hold significant promise for advancing fundamental concepts within angiogenesis, tubulogenesis, and ex vivo vascular formation in regenerative medicine [1,2,6,10].

Maintaining functional circulatory networks within microfluidic devices is essential to study the responsiveness of microvasculature to biomechanical signals and changes in the microenvironment [11,12]. Despite many significant innovations in biomimicry, the recreation of durable and stable capillaries has encountered challenges in accurately replicating native luminal diameter and vessel density *in vitro* [13,14]. While ETV2-driven ECs demonstrate branching patterns approximating native human physiology, the lumen of the “capillaries” they form are larger and vary in size [9], in contrast to the typical 7–10 µm capillary lumens typically found in vivo [15,16,17].

Shear stress, flow, and pressure are core biophysical parameters of every microfluidic device for vascular network formation that critically regulate cellular behavior and determine the overall performance of microfluidic vascular systems [4,12,18,19]. Changes in pressure and shear stress have been reported to activate mechanoreceptors within ECs, including Piezo1 and Piezo2 cation channels, which likely has significant effects on the vascular formation of ECs cultured in microfluidic devices [20,21,22,23]. Increases in shear stress can upregulate Notch-Cx37-p27 pathways to influence the differentiation of vessels into arteries and arterioles [24].

Hydrostatic pressure remains one of the most significant parameters due to its potential importance in controlling vasculature growth, remodeling, and function as well as its subsequent pressure-induced flow as well as interaction with biomechanical factors [25,26,27,28,29]. In the existing framework of the mechanobiology of angiogenesis, constant hydrostatic pressure modulates early tubulogenic activity in a magnitude-dependent manner, which can involve activated Ras-ERK/aquaporin-1 and YAP signaling [25,30,31]. Past studies commonly employ syringe pumps and gas chambers to tightly control hydrostatic pressure and/or flow microfluidic devices, creating closed perfusion systems [3,30,31]. Gravity-driven hydrostatic pressure offers a simple, stable, and physiologically relevant method for applying pressure without the mechanical fluctuations associated with pump systems [25,32]. This enables the study of pressure-dependent tubulogenesis in more accessible and open microfluidic configurations. Applying constant pressure and flow to optimize the cellular environment can affect cell viability, cell cycle progression, and differentiation for experimental and clinical applications of microfluidic devices [30,33,34]. As an alternative approach, gravity-driven pressure gradients enable the simulation of dynamic microenvironments, including spatially and temporally varying flow fields characteristic of native tissues, and are marked by high flow sensitivity resulting from their dependence on hydrostatic pressure differentials. Given its simplicity and ability to generate pressure gradients without active pumping, this method is advantageous for examining shear stress, transient flow patterns, and tubulogenesis under changing biomechanical cues [35,36,37].

Monitoring hydrostatic pressure is crucial for understanding and controlling microfluidic environments. Previous studies have integrated optical or electrical pressure sensors directly into microfluidic devices to track pressure over time [38,39]. However, these embedded sensors typically demand significant hardware and software infrastructure, along with substantial modifications to interface with microfluidic platforms, often incompatible with commercially available devices. Additionally, prior systems often rely on proprietary data acquisition tools with complex software environments and specialized hardware interfaces, which can introduce steep learning curves, limit experimental flexibility, and increase overall costs—posing practical challenges for many research laboratories. In contrast, our approach leverages commercially available, off-the-shelf pressure sensors in an external configuration integrated with widely used microfluidic devices. This eliminates the need for on-chip sensor fabrication, significantly simplifying device assembly while enhancing accessibility and reproducibility. Although this off-chip configuration does not provide spatially resolved pressure data within the chip, it effectively captures inlet and outlet pressures, which are sufficient for modeling physiologically relevant pressure–flow dynamics. Furthermore, the system is open-source, cost-effective, and adaptable, making it a practical and scalable solution for a broad range of research applications.

Our research highlights how pressure gradients and pressure–flow dynamics affect the functionality and long-term stability of vasculature formed by ETV2-driven R-VECs within a microfluidic platform under controlled hydrostatic pressure gradients, specifically focusing on pressure–flow dynamics. In this manuscript, we present a novel continuous monitoring system for real-time analysis of vessel formation and network stability *in vitro*. By integrating pressure sensors into a microfluidic device that uses pressure gradients to induce tubulogenesis, we obtain direct, real-time measurements of pressure throughout key stages of vascular development. This setup allows modulation of pressure to investigate how varying flow conditions influence micro-vessel complexity and durability under low- and high-pressure environments. Extended incubation of 3D microvasculature devices with continuous monitoring provides insights into how differential pressure and flow affect vessel sprouting, maturation, functionality, and tissue viability. Ultimately, characterizing the interplay between hydrostatic pressure gradients and flow rates will help elucidate how pressure dynamics shape vessel morphology and network physiology. In addition to a critical assessment of the stages of tubulogenesis, the continuous monitoring of experiments enables early detection of malfunctions and failed experiments, allowing for necessary interventions and timely adjustments. By providing real-time insights, this proactive approach improves data reliability and improves the efficiency of network formation within the device. An understanding of pressure dynamics is essential for engineering stable, long-lasting microvasculature that accurately replicates native blood vessel architecture.

## 2. Theory and Design

**Figure 1:**
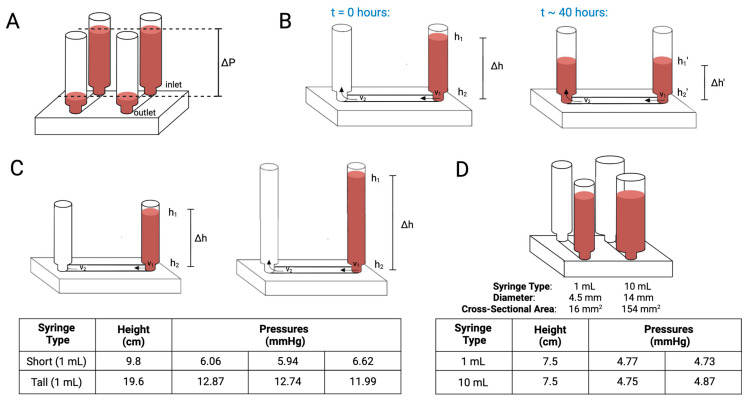
Schematic of the gravity system utilized to create a pressure gradient across the microfluidic device. (**A**) The change in pressure established between inlet and outlet syringes over the course of time. (**B**) Media equilibration over the course of 40 h as the pressure equilibrates across the channel. (**C**) The effect of reservoir height on the initial hydrostatic pressure measured at the outlet as shown. (**D**) The role of reservoir volume at constant reservoir height on initial hydrostatic pressure measured at the inlet. Two different size syringes (i.e., 1 mL, and 10 mL) measured to ensure the height of reservoir is constant. Created in BioRender. Suresh, G. (2025) https://BioRender.com/8ss6jmg accessed on 10 July 2025.

Pressure-driven systems have emerged as a viable solution for promoting flow in *in vitro* models of vascularization. Our system relies on the fundamental principles of fluid dynamics to create and maintain pressure gradients that facilitate the creation of microvasculature designed to replicate capillaries and the subsequent circulation of nutrient-rich media through them (Figure 1A,B). Central to this concept is the application of Bernoulli’s equation, which describes the relationship between pressure, velocity, and reservoir height in a fluid system.

To quantify the role of reservoir height on the hydrostatic pressure at the base of the inlet well into the microfluidic channel, a variation of the Bernoulli’s equation can be used, as shown in Equation (1). According to this formula, pressure is a direct function of height, regardless of volume and with constant fluid density (i.e., media composition) and acceleration due to gravity. Therefore, there is a direct relationship between the height of the column and the pressure as any change in height results in a corresponding proportional change in pressure, confirmed by the readings from the pressure-monitoring system (Figure 1C).

(1) P = ρgh

This was experimentally tested by setting up 1 mL and 10 mL syringes filled to the same height (7.5 cm). At this height of the fluid reservoir, there were a drastic difference in the cross-sectional areas of the syringes, and therefore, notable differences in the volumes of fluid used to establish reservoir height. However, the hydrostatic pressure taken from the base of the inlet remained the same for both conditions at the onset (i.e., t = 0), confirming the principle (Figure 1D).

The critical parameters—pressure and shear stress—within a microfluidic device, vary with velocity and time. The system under study consists of a fluid reservoir connected to a microfluidic channel, whereby over approximately 40 h, the height of the fluid in the inlet syringe decreases from $h_{1}$ to ${h_{1}}^{\prime }$ (Figure 1B). This change is a direct result of fluid flow through the device and is governed by gravity and Bernoulli’s equation where

(2) $$\Delta P=\rho g\left(h_{2}-h_{1}\right)+\frac{1}{2}\rho \left({v_{2}}^{2}-{v_{1}}^{2}\right)$$

Δ*P* is the pressure differential between two points, ρ is the fluid density, g is acceleration due to gravity, and $v_{1}$ and $v_{2}$ refer to the velocity at the inlet and outlet, respectively. Assuming a static inlet ($v_{1}$ = 0) and outlet height $h_{1}$ = 0, Equation (2) can be simplified to the following:

(3) $$\Delta P=\rho g\left(-h_{1}\right)+\frac{1}{2}\rho \left({v_{2}}^{2}\right)$$

We derive pressure as a function of both reservoir height and fluid velocity. To quantify the velocity at the outlet port within the system, we rearrange the equation as shown below:

(4) $$v_{2}={\left(\frac{2\left(\Delta P+\rho gh_{1}\right)}{\rho }\right)}^{\frac{1}{2}}$$

The relationship between time, pressure, and velocity is key to understanding the overall dynamics of the microfluidic flow system. Two relationships are evident as time (t) increases: Δh(t) is always less than Δh(0) and $h_{1}$(t) is always less than $h_{1}$(0), assuming the fluid flow remains continuous and there is no additional input of fluid to the reservoir (Figure 1A). Therefore, as time increases, there is depletion of fluid in the inlet reservoir over time, contributing to a steady decrease in both the height difference (Δh) and the initial reservoir height ($h_{1}$), resulting in decreased $v_{2}$ into the outlet, as per Equation (3) and therefore decreased shear stress (*τ*). Experimentation with different sized syringes and initial $h_{1}$ varies $v_{2}$ and *τ*.

By understanding and applying Bernoulli’s equation, we can effectively manipulate flow characteristics in the pursuit of optimizing conditions for neovascularization.

**Figure 2:**
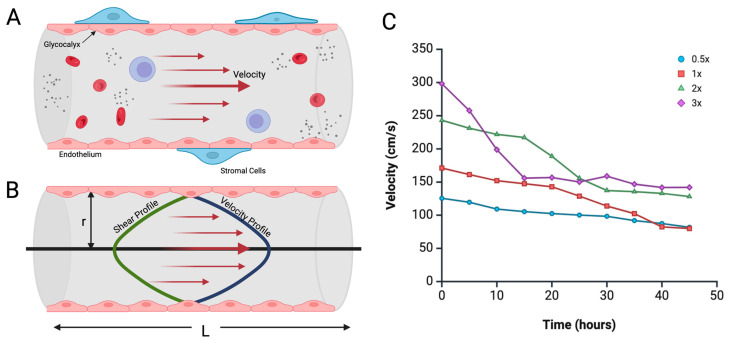
Representation of the schema of shear dynamics and the velocity profile in a microfluidic channel. (**A**) Physiologic blood vessel modeled as a cylinder to derive equations for flow and velocity of blood. (**B**) Distribution of velocity and shear profiles for a cylindrical tube, keeping in mind that the shear stress acts against the flow, including equation variables such as *r*. (**C**) Relationship between the theoretical outlet velocity ($v_{2}$) of media and time for different pressure conditions in a microfluidic channel, using recorded pressure values in Equation (4). Created in BioRender. Suresh, G. (2025) https://BioRender.com/menc6ea accessed on 10 July 2025.

To understand the mechanical forces acting on the ECs within the microfluidic channel, it is essential to calculate shear stress (*τ*), which is the tangential force exerted by the flowing media on the walls of the micro-vessels (Figure 2A,B). In microvascular systems, shear stress is a critical parameter influencing EC behavior, including alignment, permeability, and angiogenic signaling [40].

Shear stress is directly related to fluid viscosity (*μ*), flow rate (*Q*), and the geometric characteristics of the channel through which the fluid moves. For a cylindrical channel, the wall shear stress can be approximated by the following equation:

(5) $$\tau \;=\;\frac{4\mu Q}{\pi r^{3}}$$

where *τ* is shear stress, *μ* is the dynamic viscosity of the media, *Q* is the volumetric flow rate, and *r* is the radius of the circular cross-section of the cylindrical vessel.

The flow rate, *Q*, can be derived from the velocity, $v_{2}$, obtained through Bernoulli’s principle (Equation (4)), assuming laminar flow and uniform velocity across the cross-section of the outlet vessel.

(6) $$Q=Av_{2}=(\pi r^{2})v_{2}$$

Substituting Equation (6) into Equation (5), we derive an equation for shear stress as a function of velocity:

(7) $$\tau =\frac{4\mu v_{2}}{r}$$

To express shear stress in terms of pressure changes and reservoir height, we substitute $v_{2}$ from Equation (4):

(8) $$\tau =\frac{4\mu }{r}{\left(\frac{2\left(\Delta P+\rho gh_{1}\right)}{\rho }\right)}^{\frac{1}{2}}$$

This equation links shear stress to the system’s pressure gradient, reservoir height, and media viscosity—all parameters that can be experimentally controlled. As the reservoir height, $h_{1}$, decreases due to continuous fluid outflow, both the pressure and resulting velocity, $v_{2}$, decrease, which leads to a gradual decline in shear stress within the microvasculature.

These experimentally controlled variables may be leveraged to predict and model shear stress within the engineered microvasculature, enhancing the ability to extrapolate data to compare and eventually replicate physiological conditions. For instance, velocities within the inlet and outlet of the device cannot be measured without complicated, time-intensive flow assays. Using recorded pressures, Equation (4) enables the calculation of velocities over time under different pressure gradients (Figure 2C). Monitoring these changes over time allows us to model the evolving biomechanical environment experienced by the R-VECs, providing deeper insights into time-dependent vascular remodeling and microvascular stability. It is also important to consider additional parameters, such as changes in resistance and vessel branching, that can influence estimated values of shear stress, velocity, flow, and pressure.

## 3. Materials and Methods

### 3.1. Biological Setup

#### 3.1.1. Cell Culture

Approval for the use of discarded left-over Human Umbilical Vein Endothelial Cells (HUVECs) was granted by the Weill Cornell Medicine Institutional Review Board (IRB). For R-VEC generation from HUVECs, cells were transduced with lenti-PGK-ETV2 or an empty lentiviral vector at passage 1–2 [9]. R-VECs were isolated in the lab using a collagenase-based digestion method [41]. Cells were cultured in tissue dishes pre-coated with 0.2% gelatin and maintained in complete EC medium as specified, consisting of 400 mL of M199 (Fisher Scientific, Waltham, MA, USA, SH30253FS), 100 mL of heat-inactivated fetal bovine serum (FBS) (Corning, Corning, NY, USA), 7.5 mL of HEPES (Corning, Corning, NY, USA, 25-060-Cl), 1X Antibiotic–Antimycotic (Gibco, Waltham, MA, USA, 15240-062), 5 mL of Glutamax (Thermo Fisher Scientific, Waltham, MA, USA, 35050061), 100 μg/mL of heparin (Sigma-Aldrich, St. Louis, MO, USA, H3393), and growth factors: 10 ng/mL of bFGF (Peprotech, Cranbury, NJ, USA, 100-18B), 10 ng/mL of EGF (Peprotech, Cranbury, NJ, USA, AF-100-15), and 10 ng/mL of IGF (Peprotech, Crabury, NJ, USA, AF-100-11). Cells were washed with phosphate-buffered saline (PBS), then either re-fed or digested with Accutase (Corning, Corning, NY, USA, 25-058-CI) before being passaged into gelatin-coated plates as needed. HUVECs from more than five different isolations were used in the experiments. Human intestinal adenocarcinoma organoids were obtained from Weill Cornell Precision Medicine [42]. Organoids underwent transduction using an mCherry lentivirus. To facilitate propagation, organoids were digested using TrypLE Select (Thermo Fisher Scientific, Waltham, MA, USA) with additional supplements tailored to preserve organ specificity. The organoids were then cultured in a suitable medium (1:1, IntesticultTM OGM Human Basal Medium: Organoid Supplement, STEMCELL Technologies, Vancouver, BC, Canada, 100-0190, 100-0191) and maintained in 12-well low-attachment plates, embedded within 30 μL Matrigel droplets.

#### 3.1.2. Cell Seeding Within 3D Vessel Formation Assay

**Figure 3:**
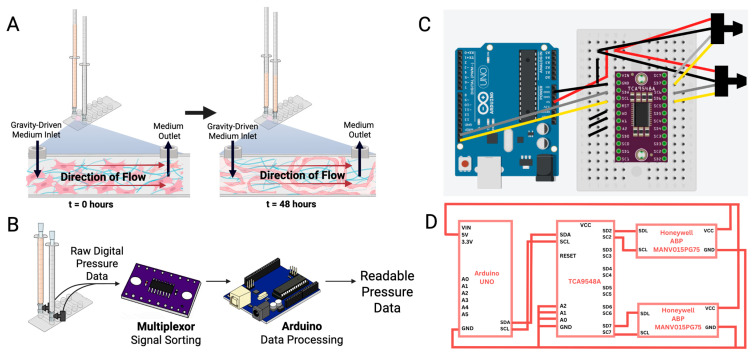
Experimental setup and Arduino-based multiplexing system and associated data flow of the system. (**A**) Schematic of pressure-driven vascularization within the microfluidic channel. (**B**) Information flow of the pressure-monitoring system and its incorporation into the microfluidic platform. (**C**) Wiring and connection diagram using an Arduino, TCA9548A multiplexor, and ABP Pressure Sensors via the I2C communication protocol. (**D**) Circuit block diagram of pressure-system including pinouts of the microcontroller, multiplexor, and sensors. Created in BioRender. Suresh, G. (2025) https://BioRender.com/v76ya5d accessed on 10 July 2025.

R-VECs digested with Accutase were collected. Cell suspensions were centrifuged at 1200 RPM for 4 min, supernatant was aspirated, and the acquired cell pellet was resuspended in 1 mL of neutralization buffer consisting of at least 10% FBS. Cells were counted using a hemocytometer and 200,000 cells (7.5 × 10^6^ cells/mL) were aliquoted into each centrifuge tube. This new cell suspension was centrifuged again, supernatant aspirated, and pellet resuspended in 36 µL of a fibrinogen solution (0.16% human fibrinogen (Millipore Sigma, Burlington, MA, USA, 341578), 0.83% bovine fibrinogen (Sigma-Aldrich, St. Louis, MO, USA, F8630), 99% X-VIVO (Lonza, Walkersville, MD, USA, 02-060Q). For colon organoid co-culture experiments, 50–100 tumor organoids and additional 50,000 R-VECs were added to the fibrinogen re-suspension, resulting in an approximate seeding ratio of 1:2500 (tumor organoids to R-VECs). Promptly, 4 µL of thrombin solution (10% bovine thrombin (Sigma-Aldrich, St. Louis, MO, USA, T4648), 90% X-VIVO) was added to the mixture, and 30 µL of the solution was seeded into a lane of an Ibidi µ-Slide VI 0.4 device (Ibidi, Fitchburg, WI, USA, 80606) via the inlet port. Once all lanes were seeded, the device was covered and incubated for 10 min at 37 °C for fibrin polymerization. After the incubation period, each inlet and outlet port was supplemented with EC tube media (StemSpan (STEMCELL Technologies, Vancouver, BC, Canada), 10% KnockOut Serum Replacement (Gibco, Waltham, MA, USA, 10828028), 1X HEPES, 1X Antibiotic–Antimycotic, 100 μg/mL heparin, 10 ng/mL bFGF, and 1 μg/mL Aprotinin (Millipore Sigma, St. Louis, MO, USA, A1153)), and air bubbles were evacuated before the addition of 1 mL syringes on each side. To test the role of high pressure on vascularization, a 1 mL unfiltered serological pipette was inserted into the 1 mL syringe to increase the height of the inlet while keeping the volume constant at 1 mL. For volume studies, 2 different syringe types (1 mL, 10 mL) were used. Syringes were then filled with the appropriate volumes of tube media and complete devices were incubated at 37 °C (Figure 3A).

#### 3.1.3. Immunofluorescent Staining

After aspiration of old tube media from the inlet and outlet syringes of the microfluidic devices, 500 µL of fresh tube media containing 1.0 µL of VE-cadherin monoclonal antibody (Alexa FluorTM 647, Thermo Fisher Scientific Waltham, MA, USA, MA5-44146) or CD31 monoclonal antibody (Brilliant Violet 605™, BioLegend San Diego, CA, USA, 303124) was added to each inlet. The antibody solution was incubated at 37 °C until 200 µL of flow was observed at the outlet reservoir, ensuring adequate antibody distribution. The tubes were then washed once with PBS and fixed with 4% paraformaldehyde (PFA) at room temperature until 200 µL of flow was established. After three washes with PBS (5 min each), samples were prepared for imaging via confocal microscopy using an Axiom Optics NL5 line-scanning confocal microscope. Any further image processing or analysis was performed with ImageJ (version 1.54p) or Imaris (version 10.2) including calculations of lumen diameter and vessel density.

### 3.2. Data Acquisition and Analysis

#### 3.2.1. Pressure Sensor Selection

The Honeywell ABP MANV015PG75 sensor (Honeywell, Charlotte, NC, USA) was selected out of a large pool of analog and digital sensors due to its availability, reusability, and pertinent specifications. The amplified basic sensors were constructed with a single axial barbed port specifically made for liquid media environments. With high sensitivity and a range for measuring 0 to 15 PSI, this sensor was best for integration into the small, liquid-pressurized environment and the specific dimensions of the syringes used in the experiment. The surface mount technology, 14-bit digital data collection, and 5.0 VDC power supply allowed for easy use with an Arduino microcontroller using its designated I2C communication line [43].

#### 3.2.2. Pressure Sensor Calibration and Signal Conditioning

The pressure-monitoring system utilized digital pressure sensors (Honeywell, ABP MANV015PG75). To ensure sterility and reusability, the sensor barbs were washed with ethanol and exposed to UV light before use. To calibrate the sensors, known pressures were applied to the sensor’s barbed port, and the resulting output signal was manually processed, measured, and recorded. Sensor outputs were compared to known pressures using a line of best fit (Figure S1). A Honeywell ABP-tested library was subsequently employed to validate that the sensor signal output matched the manually processed data. For integration with the microfluidic device, the sensors were modified by drilling a 0.08-inch diameter hole into a male-to-female luer coupler (Avantar Nylon MFLX45501-82). The axial port of the sensor was inserted into the hole and secured with lab-grade epoxy to ensure a leak-proof connection. The luer connector was then attached to the microfluidic device, and calibration was further validated by measuring known pressures generated by varying media column heights in syringes. To detect leaks, dye testing was conducted under static pressure for over an hour. Any noticeable changes in pressure or leakage of dye were addressed by reinforcing the epoxy seal and allowing for extended curing time as necessary.

#### 3.2.3. Pressure Signal Acquisition

Pressure signals were acquired using an Arduino UNO Rev3 board (Arduino SA, Monza, Italy) and ATmega328P-based microcontroller (Microchip Technology Inc., Chandler, AZ, USA). The pressure sensors communicated via an I2C channel in the Arduino and the conditioned signals were read by the microcontroller in 5 min increments (Figure 3B–D). Due to identical I2C addresses on the sensors, a TCA948a multiplexor was used to differentiate and sort the input and output signals from the sensors. The acquired pressure data was transmitted directly to a laptop computer implementing the serial monitor at a baud rate of 9600 bits per second via a USB connection. Each pressure sensor’s time of signal was recorded at each point of data acquisition. Using a flask server, pressure sensor data was displayed on an HTML page via a public server for remote monitoring and recording. After the experiment, the data was transferred to a Python (version 3.9.7) program using regular expressions to parse and format the data for analysis and visualization (Figure S2, Code S1, Code S2, Code S3).

#### 3.2.4. Incubator Camera Setup

During all vessel formation experiments, a camera (Arducam 1080P IMX291, Arducam, Guangzhou, China) was placed inside the incubator to monitor experimental setup. Images were captured every 30 min using the Autoscreenshot application to track changes in column height (Figure S3). Flow rates were determined by calculating the displaced volume, measured as the drop in reservoir height divided by elapsed time.

#### 3.2.5. Programming Code

Two primary code segments were implemented for comprehensive pressure monitoring, encompassing both full-system data acquisition and real-time remote visualization. The first code segment facilitates communication between an Arduino board and the Honeywell ABP pressure sensors using Wire and Serial communication protocols. The sensors were connected to the Arduino via a multiplexer, enabling data collection through I2C communication. The system was configured to sample pressure readings at regular intervals (set to 5 min). The sensor was initialized with its physical measuring parameters and used a function that reads the respective port of the multiplexor to communicate and gather data via I2C communication. The raw data was then processed and converted to the correct units to be displayed on the serial monitor (Figure 3). The second code segment enables real-time monitoring by displaying the measured pressure data on a publicly accessible HTML webpage. This program was run concurrently with the local serial monitoring system and utilized Flask to host a web interface. By serving the processed pressure data through a Flask-based web application, external devices could access and monitor the system remotely (Code S1).

## 4. Results

In this study, we designed and developed a real-time pressure-monitoring system to observe and quantify pressure dynamics during vascularization in a microfluidic platform. The goal was to replicate physiological conditions that mimic in vivo vasculature. Our findings demonstrate that the pressure exerted on R-VECs within the platform is primarily influenced by the height of the inlet reservoir, rather than the total fluid volume (Figure 1C,D).

### 4.1. Pressure Measurement and Profile Analysis

**Figure 4:**
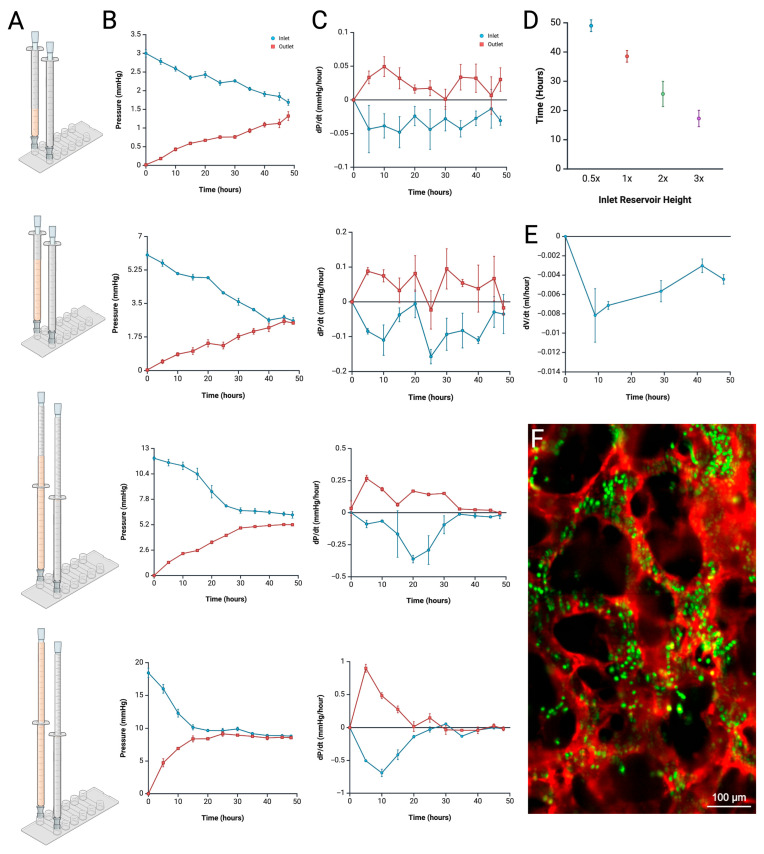
Pressure measurement and profile analysis of experimental conditions. (**A**) Inlet reservoir heights at t = 0 h of experimental conditions. (**B**) Averaged pressure sensor measurement recorded over 48 h for 0.5×, 1×, 2×, and 3× media reservoir heights at the inlet and outlet of all channels (n = 3 per condition). Data was recorded every 5 min and averaged to provide data points in 5 h intervals for clarity. Blue lines represent pressure profiles of the inlet reservoir while red lines represent pressure profiles of the outlet reservoir. Error bars represent standard error. (**C**) Analysis of change in pressure over time (dP/dt) for each experimental condition. (**D**) Equilibrium time points corresponding to different inlet reservoir heights (0.5×, 1×, 2×, and 3×). (**E**) Rate of volume change over time (dV/dt) of 0.5× reservoir height. (**F**) Engineered lumenized vascular network after 48 h of 1× gravity treatment with 6 µm beads (SPHERO™ Fluorescent Nile Red Particles, Spherotech Lake Forest, IL, USA, FP-6056-2) demonstrating effortlessly flow through vasculature (Red is CD-31 fluorescent staining, green is fluorescent beads, scale is 100 µm). See Video S1 for full video of red blood cell flow. Created in BioRender. Suresh, G. (2025) https://BioRender.com/gu4ohij accessed on 10 July 2025.

Starting at various inlet reservoir heights, inlet and outlet pressures were dynamically measured over time to equilibration. As the initial reservoir height increased (0.5×, 1×, 2×, and 3×), the inlet port pressure rose (to 3.0, 6.0, 12.0, or 18.0 mmHg, respectively) (Figure 4A,B). To quantify the rate of change in pressure over time (dP/dt, mmHg/h) for each condition, derivate graphs were plotted. Conditions varied slightly in their temporal pressure gradients, yet the initial change in pressure consistently increased as the initial reservoir pressures rose (Figure 4C). Remarkably, time to device equilibration shortened from 48 h to 15 h as reservoir height increased from 0.5× to 3× (Figure 4D). Images from the incubator camera system were utilized as another method to monitor changes in column height, and thus volume, over time. This enabled the calculation of volumetric flow through the device and validated the dP/dt flow calculations from pressure measurements (Figure 4E).

### 4.2. Anomaly Detection from Pressure Profile Analysis

**Figure 5:**
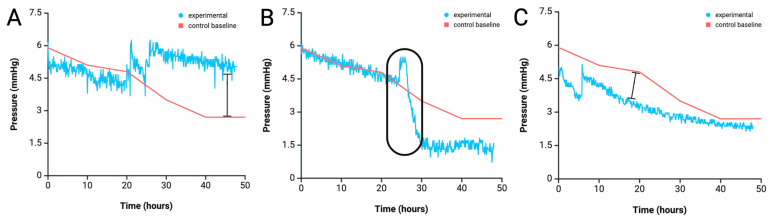
Detection of operational anomalies using pressure dynamics analysis. (**A**) Inlet pressure remains relatively stable, fluctuating between 4 and 6 mmHg. (**B**) A spike occurs at 25 h, followed by a 4.5 mmHg drop over the next 5 h. (**C**) A spike is observed at 7 h, followed by a gradual decrease from ~5 mmHg to stabilization near 3 mmHg. Red lines represent baseline pressure profiles from a 1× inlet reservoir height. Blue lines represent experiments exhibiting pressure irregularities. Created in BioRender. Suresh, G. (2025) https://BioRender.com/l5cwcpx accessed on 10 July 2025.

While a gradual decline in inlet pressures is expected as the device equilibrates, this real-time pressure-monitoring system can provide detailed insight into experimental status and possible malfunctions over time. In successful 1× height experiments, pressure should gradually stabilize around 3 mmHg by the 40 h mark without any drastic drops (Figure 4B). However, patterns such as a stable pressure with no fluctuations, sharp peaks followed by sudden drops, or early stabilization can indicate abnormalities and reduced experimental integrity (Figure 5A–C).

### 4.3. Duration of Pressure Exposure Influencing Vessel Dilation

**Figure 6:**
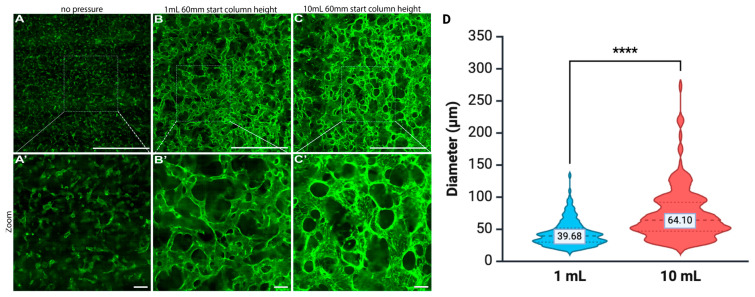
Pressure gradients promote tubulogenesis with duration of pressure exposure influencing vessel dilation. (**A**) Representative images of vessel formation with no pressure gradient. (**B**) Vessel formation using a 1 mL syringe barrel with an initial 60 mm fluid height at the inlet. (**C**) Vessel formation using a 10 mL syringe barrel with a net 60 mm fluid height at the inlet (scale bar = 1 mm). (**A’**–**C’**) Higher magnification images (scale bar = 100 µm and green is VE-Cadherin fluorescent staining). (**D**) Vessel diameters are shown for the latter two conditions. A total of 200 measurements were taken for the 1 mL and 10 mL setups each, with each condition replicated twice, displaying the median value. Statistical significance was assessed using a Kruskal–Wallis test (with **** *p* < 0.0001). Created in BioRender. Suresh, G. (2025) https://BioRender.com/5ojnznv.

To demonstrate the requirement of a pressure gradient for vessel formation, 0.5 mL of medium was placed into the inlet syringe and an equal volume to the outlet syringe (1 mL total), providing a zero-pressure gradient over the microfluidic channel. After 48 h, vessel formation was not observed (Figure 6A). However, by establishing a pressure gradient with 1 or 10 mL media in the inlet syringe only, vessel formation occurred within the same 48 h period (Figure 6B,C). To further characterize the role of pressure duration on vessel formation, a larger inlet volume of 10 mL was introduced into a 10 mL syringe barrel up to the 60 mm fluid column height. In the standard 1 mL syringe setup, 500 µL of media perfused before full equilibrium was reached as the net pressure gradient decreased from 60 mm to 0 mm, whereas in the 10 mL syringe setup, after 500 µL was perfused the difference in column height was only 3 mm, decreasing from 60 mm to 57 mm. Resulting vessel diameters were observed in the 10 mL compared to the 1 mL setup, with the median lumen diameter of 39.68 µm in the standard 1 mL condition to 64.10 µm in the 10 mL condition, which indicated that prolonged exposure to higher pressures during tubulogenesis affects the overall vessel morphology, namely yielding a grossly dilated vasculature (Figure 6D).

### 4.4. Tumoroid Co-Culture Pressure Profile Analysis

**Figure 7:**
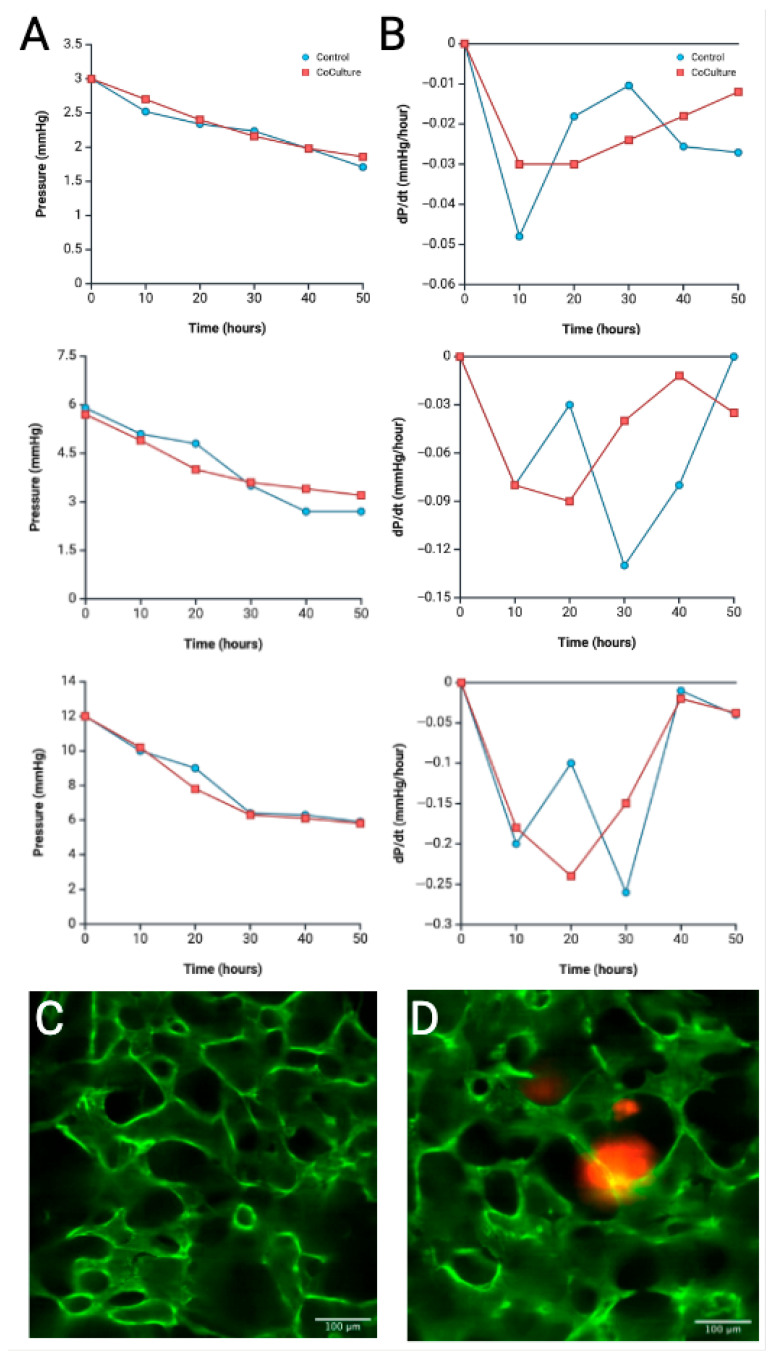
Pressure measurement and dP/dt profiles in a co-culture of R-VEC and colon organoid in a microfluidic device. (**A**) Pressure dynamics of the inlet reservoir over 48 h in co-culture vs. control of 0.5×, 1×, and 2× experimental conditions (top to bottom). (**B**) Analysis of change in pressure over time of each experimental condition over the course of the 48 h period. Blue lines represent control experiments and red lines represent the co-culture experiments. (**C**) 2D confocal reconstruction of control device after 48 h of 1 mL reservoir treatment. (**D**) 2D confocal reconstruction of colon adenocarcinoma co-culture device under 1 mL reservoir treatment (Green is VE-Cadherin fluorescent staining, red is mCherry-stained colon adenocarcinoma tumoroid, scale is 100 µm). Created in BioRender. Suresh, G. (2025) https://BioRender.com/1aom755 accessed on 10 July 2025.

Given that the microfluidic system supports both vascularization and co-culture, we sought to characterize pressure dynamics within an organ-on-chip platform. R-VECs were co-cultured with colon adenocarcinoma organoids in the device, and corresponding pressure recordings and associated dP/dt profiles were obtained. Pressure dynamics were analyzed across three reservoir height conditions of 0.5×, 1×, and 2× and compared to R-VEC only control experiments (Figure 7A,B).

## 5. Discussion

This study introduces a low-cost, customizable, real-time pressure monitoring system designed to overcome major limitations in existing commercial solutions used in microfluidic vascularization platforms. Commercial devices such as ElveFlow’s MFP/MPS sensors and Fluigent’s Pressure Unit are primarily configured for in-line pressure monitoring and often require extensive hardware, proprietary software, or significant system modifications to operate within gravity-driven setups common in organ-on-chip applications. These constraints not only increase costs but also reduce flexibility, posing accessibility challenges for many research environments.

In contrast, our Arduino-compatible platform, integrated with a Flask-based web interface, offers a versatile, open-source solution that supports remote monitoring and broader system interoperability. The inclusion of camera monitoring further enhances usability by allowing real-time visual validation of pressure and flow conditions. Together, these features improve experimental control, improve data accessibility, and support reproducibility—critical for the reliable formation of physiologically relevant vascular networks *in vitro*.

Vessel formation (tubulogenesis) within the microfluidic chip involves at least two distinct phases: an initial stage characterized by passive diffusion of nutrients and cytokines from the culture media, followed by a lumenization event that marks the onset of active perfusion. The bifurcation point, defined by lumen formation, is critical as it separates the diffusion-dominated phase from flow-driven vascular development. Pressure plays a central role in the early stage, facilitating interstitial transport and establishing the mechanical cues necessary for endothelial organization and network formation

Using our remote monitoring system, we continuously tracked hydrostatic pressure and its rate of change (dP/dt) over a 48 h period across various microfluidic pressure gradients (6–18 mmHg). The data revealed an inverse relationship between inlet reservoir height and time to reach pressure equilibrium, suggesting that higher media columns accelerate initial pressure changes (dP/dt) and corresponding flow rates (dH/dt) (Figure 4A–E). However, the elevated flow observed at higher pressures may partially result from structural artifacts, such as the formation of non-physiological channels or matrix contractions caused by elevated mechanical stress. These findings imply that reservoir height influences not only fluid dynamics but also vessel stability, potentially affecting branching architecture, overall resistance, and long-term viability.

In addition to detecting equilibration time, the pressure monitoring system can help users to detect problems in their experiments and deduce problems, saving researchers time and resources. Experiments with constant pressure at the inlet compared to a successful experiment of that height can point to non-vascularizing cells as well as missing growth factors in the media, or incorrect ratio of reagents to create the fibrin matrix (Figure 5A). Improper technique when loading syringes with media can leads to air bubbles which can ultimately lead to leaks from the inlet reservoir or pressure sensor. Air bubbles induced leaks typically manifest as sharp pressure spikes in the inlet reservoir, followed by a rapid drop and stabilization within approximately 5 h (Figure 5B). Rapid decreases in pressure at the inlet can either be leaks or contraction of fibrin. However, equilibration around the normal corresponding equilibration pressure of that height (half of the initial inlet pressure) can indicate the rapid decline was due to contraction in the fibrin and no additional leaks (Figure 5C). Analysis of real-time pressure data from ongoing experiments can increase experimental efficiency and yield valuable insights for optimizing dynamic systems.

Microvasculature formation and tubulogenesis is a multifaceted process dependent not only on pressure but also other physical parameters including flow dynamics. Bernoulli’s principle confirms that initial pressure is a function of height (Figure 1C,D), but the duration of pressure is another significant factor to consider. When initial pressures were held constant, but volume was increased by a factor of 10, there was nearly two-fold expansion in lumen diameter, which underscores the need for further investigation into the relationship between prolonged pressure exposure, and therefore subsequent volumetric flow, and its effects on vessel morphology, particularly in terms of vessel structural adaptation and long-term stability (Figure 6). Further investigation is needed to characterize how elevated pressures and pressure durations as well as the interplay between both parameters reshape microvascular geometry and whether activation of mechano-transduction pathways contributes to these effects.

Understanding these dynamics is critical for optimizing system design and ensuring accurate pressure monitoring. Such insights may ultimately inform the development of more stable and physiologically relevant vascular models for long-term studies *in vitro*, with significant implications for microfluidic co-culture practices in regenerative medicine [9]. In this study, pressure dynamics were evaluated over 48 h in a co-culture system containing R-VECs and colon adenocarcinoma organoids, serving as a representative example of disease modeling; the goal of integrating R-VECs and microfluidic platforms (Figure 7). Colon adenocarcinomas were chosen due to prior research on secretion of pro-angiogenic factors and effects on modulating EC behavior [44,45,46]. Based on the increased cell density and anticipated secretion of angiocrine factors by the tumor organoids, we hypothesized that co-culture lanes would reach pressure equilibrium more rapidly. Contrary to the expectations, however, the pressure profiles closely resembled those of the control lanes. This unexpected similarity in pressure trends suggests that other factors, such as increased matrix resistance, delayed endothelial remodeling, patient-specific tumor phenotype, or competition between cell populations, may offset the predicted effects. These observations highlight the complexity of co-culture environments and underscore the need for further investigation elucidate the interplay between cellular, structural, and mechanical variables in such systems.

The experimental validation of Bernoulli’s equation as it applies to this microfluidic vascularization model reinforces the fundamental role of fluid dynamics in both the formation and maintenance of viable vascular networks. The ability to empirically confirm that pressure is a function of height, independent of volume, underscores the precision with which this system can replicate the mechanical forces present in in vivo environments (Figure 1C,D). These findings provide a rudimentary methodological framework for future mechanistic studies of the microvascular responses to fluctuating pressures and theoretical shear stress (Figure 2C). However, uncontrolled variables such as vessel branching, the resulting changes in resistance, and dynamic remodeling of vascular architecture over time must be investigated further to better understand the biomechanical environment and perfusion kinetics within the engineered vasculature. By establishing controlled pressure gradients, researchers can manipulate fluid flow dynamics, thereby facilitating the development of functional vascular networks in *in vitro* microfluidic platforms [4,47]. This approach not only mimics the physiological conditions found in living tissues but also allows for precise manipulation of pressure-modulated forces and targeted delivery of biochemical cues, further advancing the study of vascular development and adaptation.

The application of the Bernoulli principle allows researchers to predict and control flow velocities at different points within the microfluidic system, enabling more tightly regulated conditions for promoting cell survival, proliferation, adhesion, and migration. By finetuning these parameters, it becomes possible to create more precise and reliable models of vascularization, including complex, multilayered systems with multiple inlets and outlets that more closely resemble the hierarchy of vessel networks in vivo. These advancements bring us closer to developing fully functional, vascularized organ-on-a-chip models that reduce the reliance on animal testing for translational studies and hold significant promise for drug screening, tissue engineering, and the investigation of complex disease processes, including tumor angiogenesis [2,4,6].

By analyzing the effects of various flow and pressure conditions on lumen formation, this study offers critical insights for enhancing the physiological relevance of *in vitro* vascular models. This system also enables real-time monitoring of cellular responses to hemodynamic forces which are crucial in EC viability and network stability [6,8]. The system’s remarkable capacity to precisely monitor and adjust flow rates and pressure gradients could facilitate further investigation of mechanotransduction pathways known to regulate vascular differentiation and morphogenesis [20,21,22,23,24]. Furthermore, the direct correlation between pressure dynamics and vessel stability may help uncover thresholds for pressure and related variables that promote optimal cellular behavior and tissue integrity.

Future studies can expand upon this work by investigating a broader range of pressure and volume conditions to further refine the optimal parameters for vascular network formation. Incorporating multi-channel configurations could provide insights into more complex flow dynamics, while experimenting with different biomaterials could shed light on structural and biochemical properties driving vascular formation. Additionally, altering specific mechanosensory pathways could reveal critical changes in the pressure–flow relationship, offering a deeper understanding of how mechanical forces influence vascular development. Ultimately, novel approaches in the finetuning of mechanics in 3D, vascularized microfluidic systems will advance tissue engineering and regenerative medicine applications.

## 6. Conclusions

The real-time pressure-driven monitoring system represents a significant advancement in microfluidic-based vascularization, providing a robust framework to monitor the hemodynamics of a vascularized construct for both fundamental research and translational applications. The insights gained from this study will not only enhance our understanding of microvascular formation but also contribute to the development of scalable physiologically relevant vascularized models for use in tissue engineering, disease modeling, pharmacological testing, and personalized medicine.

## Data Availability

The original contributions presented in this study are included in the article/Supplementary Materials. Further inquiries can be directed to the corresponding author.

## Supplementary Materials

The following supporting information can be downloaded at https://www.mdpi.com/article/10.3390/biomimetics10080501/s1, Figure S1: Calibration curve of the pressure sensor showing the direct relationship between applied pressure and raw sensor output; Figure S2: System Architecture and Data Flow for Pressure Sensing and Visualization; Code S1: Arduino Code for Multiplexed Pressure Sensor Data Acquisition and Conversion, Code S2: Python Flask Server for Real-Time Arduino Serial Data Visualization, Code S3: Python Script for Parsing and Extracting Pressure Data from Serial Output, Figure S3: Incubator Camera Images for dV/dt Measurement, Video S1: Vascular network after 48 h of 1× gravity treatment with human red blood cells flow.

## Abbreviations

The following abbreviations are used in this manuscript:

HUVECs	Human Umbilical Vein Endothelial Cells
R-VECs	“Reset” Vascular Endothelial Cells
ECs	Endothelial Cells
