# Supplementary material for: Design and Development of a Real-Time Pressure-Driven Monitoring System for In Vitro Microvasculature Formation
Source: Biomimetics (Basel). 2025 Aug 1;10(8):501. doi: 10.3390/biomimetics10080501 (PMC12383887; doi:10.3390/biomimetics10080501)
Supplement: Supplementary file 1 [file biomimetics-10-00501-s001.zip › Supplementary Materials/Code_S2.rtf]

import serialimport timefrom flask import Flask, jsonify, render_template_stringimport threadingapp = Flask(__name__)serial_data = []  # global list variable to store the incoming serial data from Arduinotry:    ser = serial.Serial('/dev/cu.usbmodem144401', 9600)  # opening port for Arduino connection with appropriate baud rate    time.sleep(2)  # Wait for connection to establish    print("Serial port opened successfully.")except serial.SerialException as e:    print(f"Error: {e}")  # error handling if it doesn't connect    exit()def read_serial_data():    try:        if ser.in_waiting > 0:  # checks if there is data already waiting in the serial register            line = ser.readline().decode('utf-8').rstrip()  # reads the data and decodes it to a string, removing trailing whitespace            return line    except Exception as e:        print(f"Error reading serial data: {e}")  # error handling if no data waiting    return Nonedef update_serial_data():    global serial_data    while True:        data = read_serial_data()        if data:            serial_data.append(data)  # add new data to the list@app.route('/')def index():    return render_template_string('''<!DOCTYPE html><html lang="en"><head>    <meta charset="UTF-8">    <meta name="viewport" content="width=device-width, initial-scale=1.0">    <title>Arduino Serial Monitor</title>    <style>        body {            font-family: Arial, sans-serif;            display: flex;            flex-direction: column;            align-items: center;            justify-content: center;            min-height: 100vh;            margin: 0;            background-color: #f4f4f9;        }        h1, h2 {            color: #333;            margin-bottom: 10px;        }        .container {            background-color: #fff;            border: 1px solid #ddd;            border-radius: 8px;            padding: 20px;            box-shadow: 0 4px 8px rgba(0, 0, 0, 0.2);            max-width: 400px;            width: 100%;            text-align: center;            margin-bottom: 20px;        }        #currentData, #pastData {            list-style: none;            padding: 0;            margin-top: 15px;        }        #currentData li, #pastData li {            padding: 10px;            border-bottom: 1px solid #ddd;            color: #555;        }        #currentData li:last-child, #pastData li:last-child {            border-bottom: none;        }        .loading {            color: #888;            font-style: italic;        }    </style>    <script>        async function fetchData() {            try {                const response = await fetch('/data');                const data = await response.json();                                // Display current data (most recent)                const currentDataElement = document.getElementById('currentData');                currentDataElement.innerHTML = "";                if (data.length > 0) {                    const latestItem = document.createElement('LI');                    latestItem.textContent = data[data.length - 1];                    currentDataElement.appendChild(latestItem);                } else {                    currentDataElement.innerHTML = "<li class='loading'>No data available</li>";                }                                // Display past data                const pastDataElement = document.getElementById('pastData');                pastDataElement.innerHTML = "";                data.slice(0, -1).forEach(item => {                    const listItem = document.createElement('LI');                    listItem.textContent = item;                    pastDataElement.appendChild(listItem);                });            } catch (error) {                console.error('Error fetching data:', error);            }        }        setInterval(fetchData, 1000);    </script></head><body>    <div class="container">        <h1>Arduino Serial Monitor</h1>        <h2>Current Sensor Value:</h2>        <ul id="currentData" class="loading">Loading...</ul>    </div>    <div class="container">        <h2>Past Sensor Values:</h2>        <ul id="pastData" class="loading">Loading...</ul>    </div></body></html>''')@app.route('/data')def get_data():    return jsonify(serial_data)if __name__ == '__main__':    try:        ser = serial.Serial(‘insert Com Port name’, 9600, timeout=1)        time.sleep(2)  # Wait for connection to establish        print("Serial port opened successfully.")        thread = threading.Thread(target=update_serial_data)        thread.daemon = True        thread.start()        app.run(host='0.0.0.0', port=5000, debug=True)    except serial.SerialException as e:        print(f"Error: {e}")        exit()
