# Supplementary material for: Design and Development of a Real-Time Pressure-Driven Monitoring System for In Vitro Microvasculature Formation
Source: Biomimetics (Basel). 2025 Aug 1;10(8):501. doi: 10.3390/biomimetics10080501 (PMC12383887; doi:10.3390/biomimetics10080501)
Supplement: Supplementary file 1 [file biomimetics-10-00501-s001.zip › Supplementary Materials/Manuscript Supplementary Materials and Legends.pdf]

Supplementary Figures

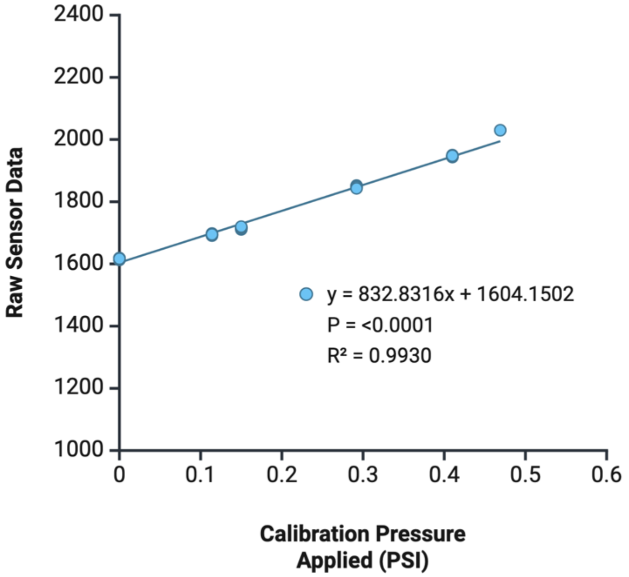

Figure S1. **Calibration curve of the pressure sensor showing the direct relationship between applied pressure and raw sensor output**. Raw sensor data was plotted against known calibration pressures ranging from 0 to 0.5 PSI (0 to 25.8575 mmHg). A strong linear correlation was observed ($R^2 = 0.9930$), with the resulting regression equation y=832.83x+1604.15, indicating the sensor's high sensitivity and reliability for pressure measurements in this range. Created in BioRender. Suresh, G. (2025) https://BioRender.com/1m67jdx

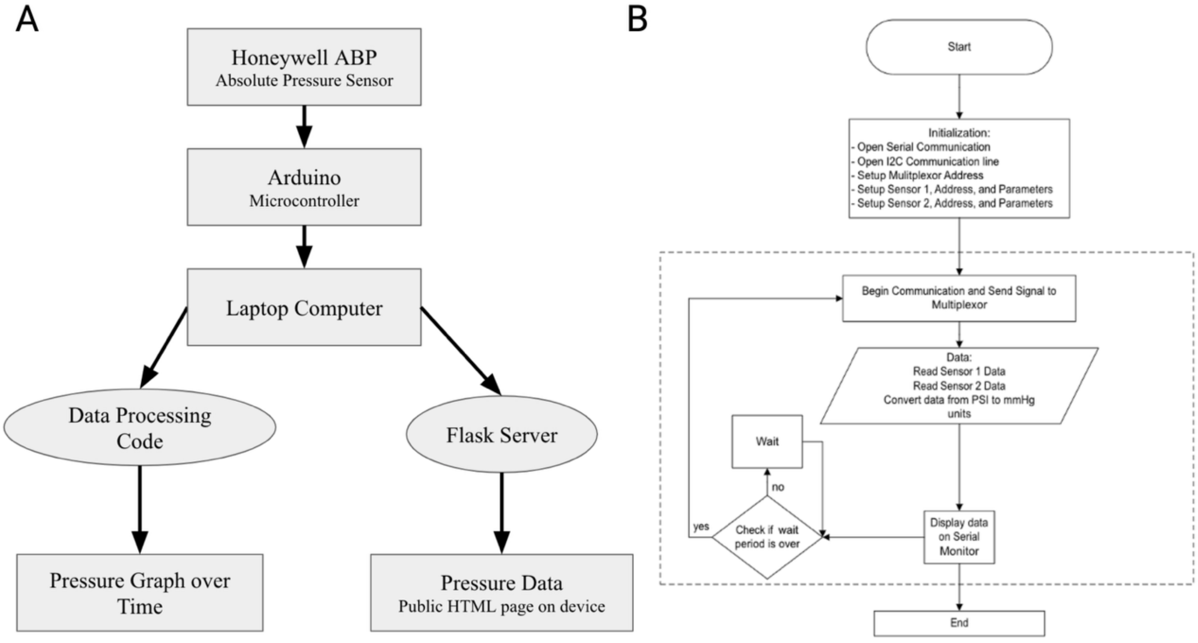

Figure S2. **System Architecture and Data Flow for Pressure Sensing and Visualization** A) Data flow of the system from data acquisition by the sensors through the microcontroller, splitting into two separate paths for remote data access and data processing. B) Logical sequence and data flow in the programming code used to acquire and process pressure data from sensors. It includes initialization of serial communication, sensor

configuration, data reading from sensors, data conversion to mmHg, and real-time display. Created in BioRender. Suresh, G. (2025) https://BioRender.com/efk9qtj

Code S1. C++ code initializes and communicates with two Honeywell ABP pressure sensors via an I2C interface using a TCA9548A multiplexer. The setup() function establishes serial and I2C connections, while the loop() function periodically reads pressure data from two sensor channels (inlet and outlet). The readSensor() function retrieves pressure values in psi and converts them to dynes/cm² for display on the serial monitor. Delays are incorporated to manage signal timing and update intervals.

Code S2. Python Flask Server for Real-Time Arduino Serial Data Visualization. Python script establishes a serial connection with an Arduino device and continuously reads pressure sensor data using a background thread. The data is stored in a global list and made accessible through a Flask web server. The / route serves a styled HTML interface that displays the most recent and past sensor readings, while the /data endpoint provides the raw data as a JSON object. The web interface uses JavaScript to asynchronously fetch and update data in real time, enabling users to monitor sensor output via a browser without additional desktop software.

Code S3. Python Script for Parsing and Extracting Pressure Data from Serial Output. Python script processes raw serial monitor output using regular expressions to extract and separate inlet and outlet pressure values reported in mmHg. The user pastes serial output data into the data variable, and the script uses re.findall() to match pressure values from specific sensor channels. Extracted values are converted to floats and printed for further analysis. This tool facilitates quick post-processing of logged pressure data from an Arduino-based sensing system.

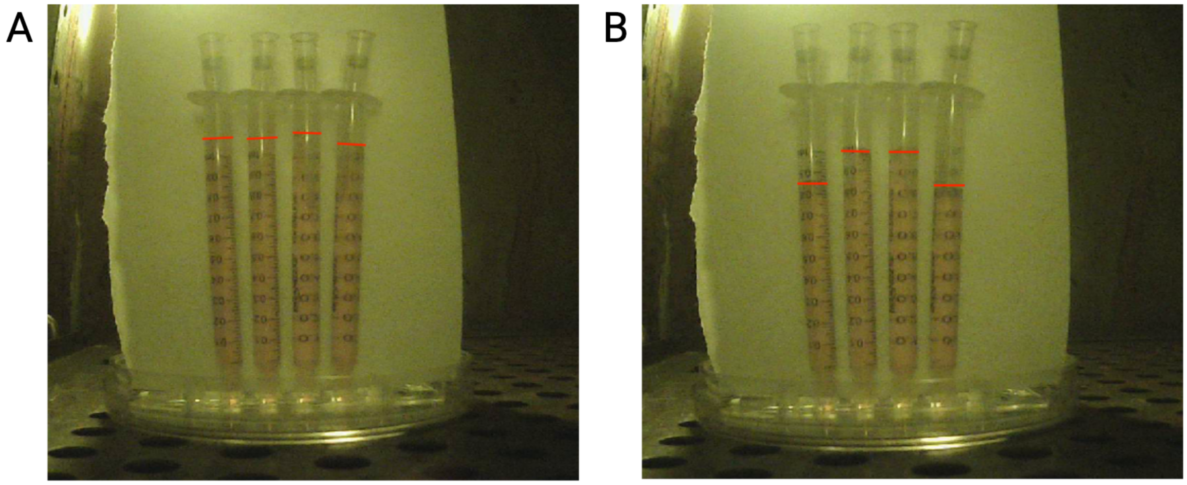

Figure S3. Incubator Camera Images for Volumetric Flow Rate (dV/dt) Measurement. (A) Initial time point showing fluid levels in four 1x reservoir height syringes at the start of the measurement period. (B) Fluid levels in the same syringes after 16.5 hours, illustrating volume displacement used to calculate dV/dt. Red

horizontal lines indicate the fluid meniscus used as the reference for volume quantification. Images were captured using a fixed-position incubator camera under constant lighting to ensure consistency in measurement. Created in BioRender. Suresh, G. (2025) https://BioRender.com/4d5w7is

Video S1. Vascular network after 48 hours of gravity treatment with human red blood cells flow. Live imaging of a microvascular network subjected to standard 1x gravity conditions after 48 hours treatment. Human red blood cells were perfused through the network to assess vessel continuity and flow. The observed flow confirms the presence of lumenized channels formed under these conditions.
