# Supplementary figures and images for: Design and Development of a Real-Time Pressure-Driven Monitoring System for In Vitro Microvasculature Formation
Source: Biomimetics (Basel). 2025 Aug 1;10(8):501. doi: 10.3390/biomimetics10080501 (PMC12383887; doi:10.3390/biomimetics10080501)

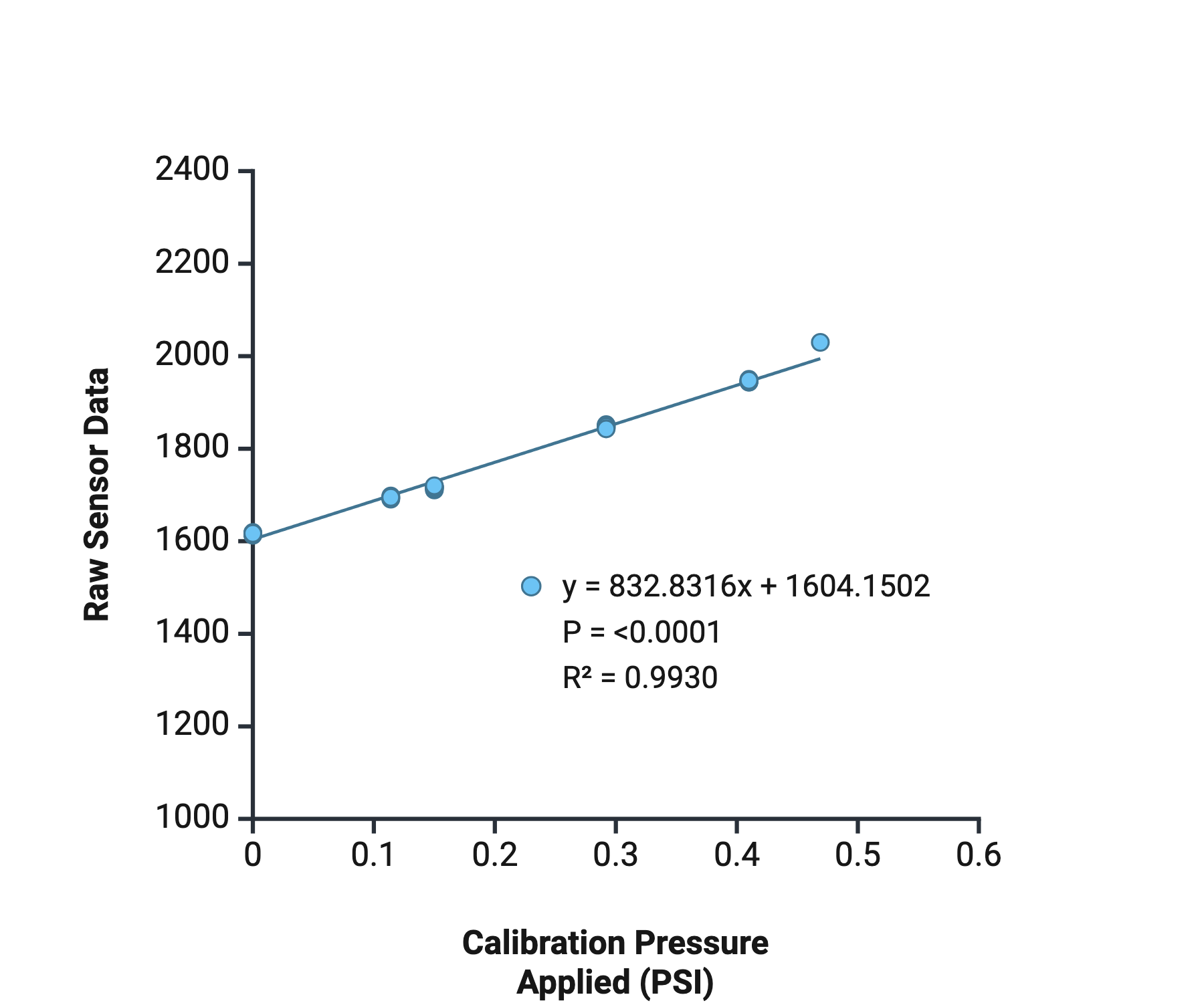

Supplement: Supplementary file 1 [file biomimetics-10-00501-s001.zip › Supplementary Materials/Figure_S1.png]

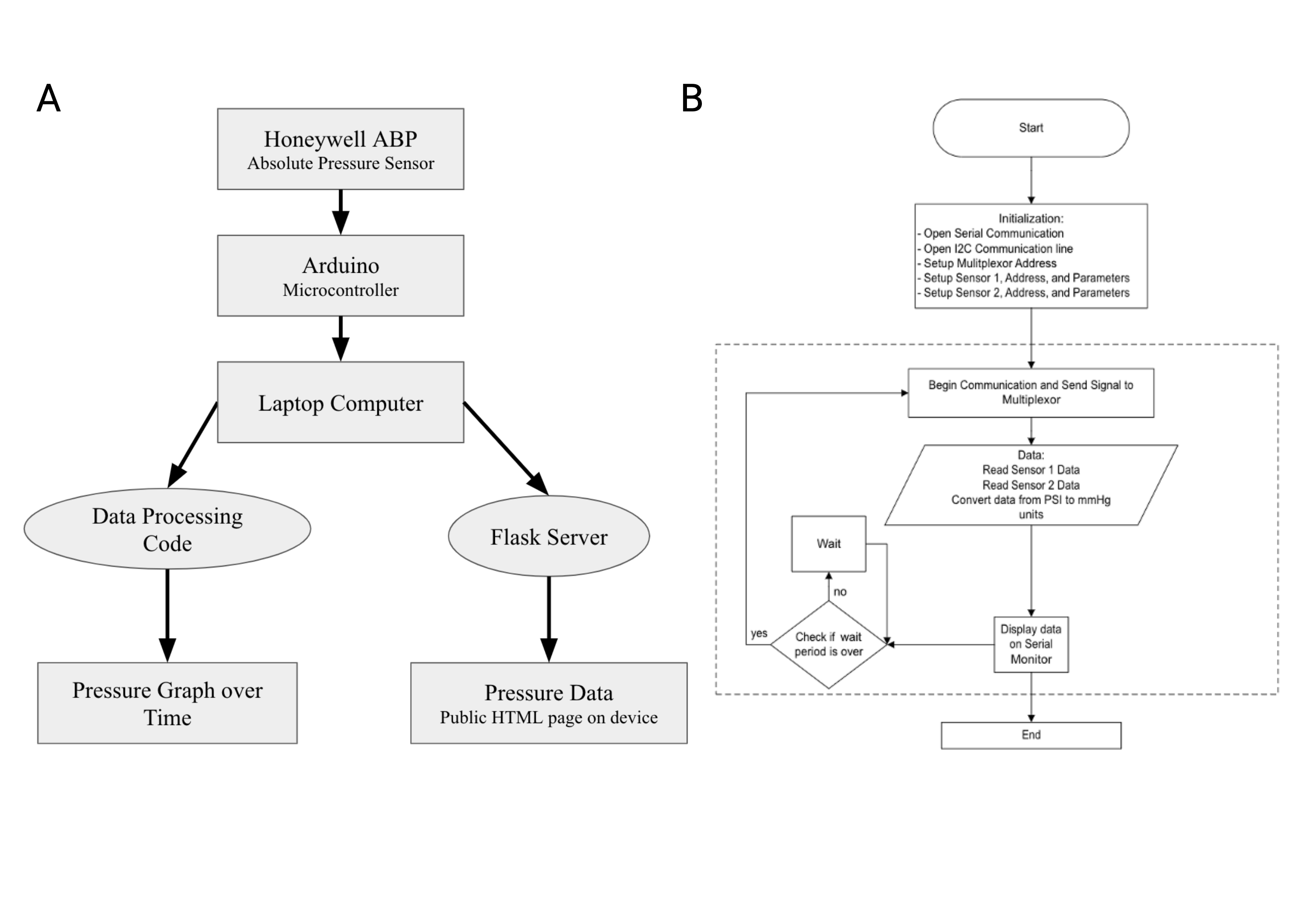

Supplement: Supplementary file 1 [file biomimetics-10-00501-s001.zip › Supplementary Materials/Figure_S2.png]

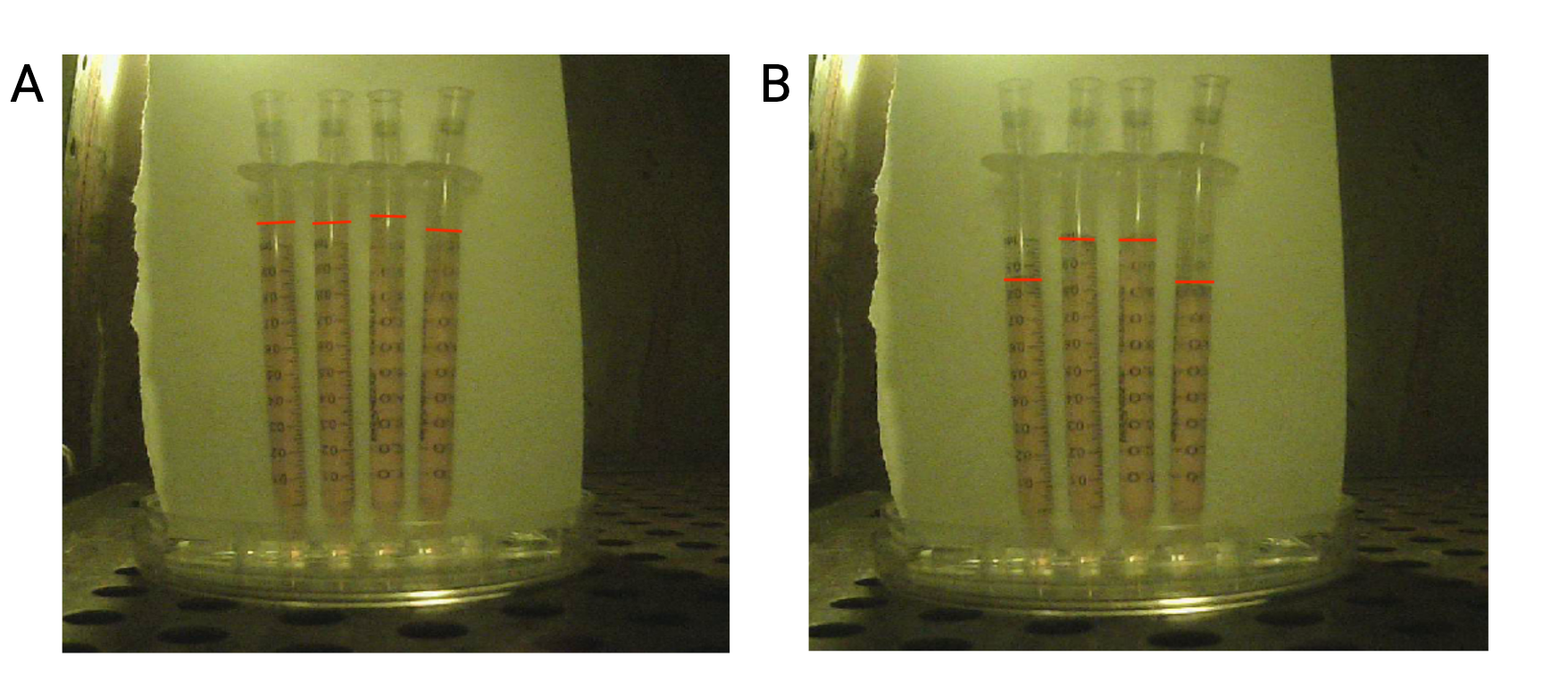

Supplement: Supplementary file 1 [file biomimetics-10-00501-s001.zip › Supplementary Materials/Figure_S3.png]
